# Clinical practice guidelines for telesurgery 2022: Committee for the promotion of remote surgery implementation, Japan Surgical Society

**DOI:** 10.1007/s00595-024-02863-5

**Published:** 2024-06-03

**Authors:** Masaki Mori, Satoshi Hirano, Kenichi Hakamada, Eiji Oki, Shigeo Urushidani, Ichiro Uyama, Masatoshi Eto, Yuma Ebihara, Kenji Kawashima, Takahiro Kanno, Masaru Kitsuregawa, Yusuke Kinugasa, Junjiro Kobayashi, Hiroshige Nakamura, Hirokazu Noshiro, Masaki Mandai, Hajime Morohashi

**Affiliations:** 1 Japan Surgical Society, Tokyo, Japan; 2 School of Medicine, Tokai University, 143 Shimokasuya, Isehara City, Kanagawa Japan; 3 Department of Gastroenterological Surgery II, Hokkaido University, Sapporo, Hokkaido Japan; 4 Department of Gastroenterological Surgery, Hirosaki University, Hirosaki, Aomori Japan; 5 Department of Surgery and Science, Graduate School of Medical Sciences, Kyushu University, Fukuoka, Japan; 6 Information Systems Architecture Science Research Division, National Institute of Informatics, Tokyo, Japan; 7 Advanced Robotic and Endoscopic Surgery, Fujita Health University, Toyoake, Aichi Japan; 8 Department of Urology, Graduate School of Medical Sciences, Kyushu University, Fukuoka, Japan; 9 Graduate School of Information Science and Technology, The University of Tokyo, Tokyo, Japan; 10 RIVERFIELD Inc, Tokyo, Japan; 11 National Institute of Informatics, The University of Tokyo, Tokyo, Japan; 12 Department of Gastrointestinal Surgery, Tokyo Medical and Dental University, Tokyo, Japan; 13 National Cerebral and Cardiovascular Center, Osaka, Japan; 14 Department of General Thoracic Surgery and Breast and Endocrine Surgery, Tottori University, Tottori, Japan; 15 Department of Gastroenteology and General Surgery, Saga University, Saga, Japan; 16 Department of Obstetrics and Gynecology, Kyoto University, Kyoto, Japan

**Keywords:** Telemedicine, Remote robotic surgery, Information technology, Communication networks, Surgical education

## Abstract

Telesurgery is expected to improve medical access in areas with limited resources, facilitate the rapid dissemination of new surgical procedures, and advance surgical education. While previously hindered by communication delays and costs, recent advancements in information technology and the emergence of new surgical robots have created an environment conducive to societal implementation. In Japan, the legal framework established in 2019 allows for remote surgical support under the supervision of an actual surgeon. The Japan Surgical Society led a collaborative effort, involving various stakeholders, to conduct social verification experiments using telesurgery, resulting in the development of a Japanese version of the “Telesurgery Guidelines” in June 2022. These guidelines outline requirements for medical teams, communication environments, robotic systems, and security measures for communication lines, as well as responsibility allocation, cost burden, and the handling of adverse events during telesurgery. In addition, they address telementoring and full telesurgery. The guidelines are expected to be revised as needed, based on the utilization of telesurgery, advancements in surgical robots, and improvements in information technology.

## Introduction

Robot-assisted surgery is rapidly gaining popularity in many surgical fields because it enables precise, minimally invasive surgery with enhanced stereoscopic magnification and full dexterity. In Japan, it was first reimbursed by national health insurance in 2012, and since then, surgical indications have been expanded to include more and more surgical fields. Recently, new domestically produced surgical robots have been approved, and many surgical robots with various functions have been introduced to the market worldwide. Robot-assisted surgery is expected to become even more commonplace in the near future.

Due to their inherent structure, surgical robots can also be used for telesurgery through information and communication devices instead of indoor cables. If telesurgery can be implemented widely in society, it is expected to improve the accessibility of medical care in areas with limited medical resources, as well as to be beneficial for the rapid diffusion of new technologies and the advancement of surgical education [1]. Therefore, to pursue this potential benefit, clinical and preclinical studies of telesurgery have been conducted in many countries, to date, including Japan [2]. In 2001, a transatlantic cholecystectomy was performed, the so-called Lindbergh operation [3], followed by a series of general surgical procedures in Canada [4]. However, these projects were abandoned in the mid-2000s due to significant communication delays, high communication costs, and the halted development of telesurgery-capable surgical robots.

In recent years, the development of high-speed communication networks and novel surgical robots have created an environment that enables social implementation of telesurgery with minimal communication delays. In Japan, a legal environment was established in 2019 to enable telesurgical support when an actual surgeon is present at the site where the surgery is being performed. It was also determined that the delivery system, technical requirements, and socio-ethical considerations for the safe implementation of telesurgical support need to be verified through social demonstration experiments, and new guidelines should be provided by the relevant academic societies. Therefore, the Japan Surgical Society, together with related academic societies, surgical robot companies along with relevant information processing companies, telecommunication companies, national research facilities, the Ministry of Internal Affairs and Communications, the Ministry of Health, Labor and Welfare, and the Japan Agency for Medical Research and Development, have launched a project for the social implementation of telesurgery and have conducted verification experiments with telesurgery in various settings and actual social environments [5–18]. These guidelines were developed as a product of this project, and the first edition was published in Japanese in June 2022. This issue of the Guidelines includes basic information not only on telesurgical support, but also on telementoring and full telesurgery, both of which have been developed in recent years. In the future, these will be revised in accordance with advances in medical technology and changing social needs.

Currently, Japan is facing a declining population, uneven regional distribution of medical resources, and a shortage of surgeons. Looking at the world as a whole, many countries and regions have scarce medical resources across a vast landmass. In the midst of these difficulties, telesurgical support is a cutting-edge technology that improves accessibility to medical care, thereby providing high-quality medical procedures over a wide area beyond the limitations of distance and space. We hope that the standardization of technical requirements and delivery systems for telesurgery through the Guidelines will contribute to humanitarian efforts in countries around the world, especially where medical resources are scarce, as well as lead to further development in medical technology and the enhancement of medical education.

1. **Chapter 1 General remarks**
2. Aim
  The purpose of the “Clinical practice guidelines for telesurgery 2022” (hereinafter referred to as the “Guidelines”) is to present appropriate standards for the systems through which telesurgery is provided and implemented, specifically when performed remotely by a supervising physician in a distant location using both surgical robots and information and communication technology.

1.2. Types of telesurgery and the scope of the Guidelines
1.2.1. Types of telesurgery
  In the Guidelines, telesurgery is classified into the following three types according to the degree of involvement of the remote physician in the surgery.

1.2.1.1. Telementoring
  The supervising surgeon remotely participates in on-site surgery where the patient is present in real time. This is a form of providing specific surgical guidance to the local surgeon through both images and voice using information and communication devices such as tablets. It also includes the use of a system to remotely project lines and arrows on a local monitor, and to supervise the entire operation by looking over and grasping the physical environment of the local operating room situation. The relationship between a remote supervisor and a local surgeon corresponds to “D to D,” or doctor-to-doctor information exchange and/or educational interactions in “the Guidelines for the Appropriate Implementation of Online Clinical Practice (https://www.mhlw.go.jp/content/001126064.pdf, In Japanese),” hereinafter referred to as the "Guidelines for Online Clinical Practice.”

1.2.1.2. Telesurgical support
  The supervising surgeon operates the surgical robot remotely to assist the local surgical team in performing the surgery as a partial surgeon or assistant. In the event of communication failure or other unforeseen circumstances, the local surgical team must be capable of completing the operation without the aid of the remote surgeon. The relationship between the remote supervising doctor and the local doctor and patient is “D to P with D” in the Guidelines for Online Clinical Practice in Japan.

1.2.1.3. Full telesurgery
  In an environment where the surgeon is not present at the patient’s side, a remote surgeon performs surgical operations with complete remote control of the surgical robot. The remote doctor–patient relationship is classified as “D to P” in the Guidelines for Online Clinical Practice. Although technically feasible, it has not been approved for implementation in Japan as of this writing.

Summary: Types of telesurgery

**Table:** 

Types	Telementoring	Telesurgical support	Full telesurgery
Outline	A remote supervisor gives verbal and graphical instructions to local surgeons remotely using tablets and other information and communication devices	A remote supervisor remotely operates the surgical robot and directly assists the local surgical team as a surgeon or assistant	A telesurgeon operates on a patient directly with complete remote control in an environment where there is no surgeon on site
Robotic operator	Local surgeon (100%)	Local surgeon and remote surgeon (Collaborative surgery is achieved by switching operating authority.)	Remote surgeon (100%)
Primary responsibility	Local surgeon	Local surgeon	Remote surgeon
Prior confirmation of liability proration	Required	Required	Not required
Emergency response	Local surgeon	Local surgeon	Local staffs other than surgeon
Types of online clinical practice in Japan*	D to D	D to P with D	D to P
Legal feasibility	Yes	Yes	No at present in Japan

*D* doctor,* P* patient

*Guidelines for the appropriate implementation of online clinical practice

1.2.2. Scope of the Guidelines
  The Guidelines apply to all three types of telesurgery. First, the main focus is telesurgical support, which requires that a supervising senior surgeon operate directly from a distance on the condition that there is a local surgeon available to respond to emergencies. Next, there is telementoring, which is expected to become increasingly popular, but needs to meet certain conditions for real-time implementation. Finally, while it is also technically feasible and included in the scope of these guidelines, full telesurgery has been included. Nonetheless, there are still many issues to be overcome from the perspective of ensuring safety in surgical performance and patient management, as well as the many requirements from a legal standpoint. Therefore, it has been difficult to implement this in Japan for the time being.

1.3. Definition of terms

The terms used in the Guidelines are defined as follows.

1.3.1. Surgical robot: A robotic surgical unit that has been approved as a surgical support system by an accredited agency. For purposes of medical reimbursement in Japan, it is described as “an endoscopic surgical support device.”
1.3.2. Robot-assisted (endoscopic) surgery: Endoscopic surgery performed using a surgical robot. The term "endoscopic surgery using endoscopic surgical support devices" is the medical reimbursement language used in Japan.
1.3.3. Remote surgery or telesurgery (full telesurgery/telesurgical support/telementoring): A remote senior surgeon providing surgery/surgical assistance/guidance between different health care facilities using information and communication technology and surgical robots.
1.3.4. Local hospital (institution): The actual facility where the patient is undergoing surgery is located.
1.3.5. Local surgeon: The surgeon who is physically present at the same facility where the patient undergoing surgery is located.
1.3.6. Local operative staff: Personnel other than the local surgeon (e.g., physicians, anesthesiologists, clinical engineers, nurses, and medical information managers, etc.) at the facility where the patient undergoing surgery is located.
1.3.7. Remote hospital (institution): The facility where a senior surgeon is enlisted to provide surgery, surgical support, and guidance, remotely, through images and voice instructions, to patients at a distant local facility utilizing information and communication technology.
1.3.8. Remote surgeon: The surgeon at a remote facility who performs telesurgery directly or provides technical support to a local surgeon by serving as the operator of a surgical robot.
1.3.9. Remote mentor: A surgeon who provides only images and audio guidance for the surgery from a remote facility, but does not perform any surgical maneuvers.
1.3.10. Remote operative staff: Personnel other than the remote surgeon/mentor (e.g., physicians, anesthesiologists, clinical engineers, nurses, and medical information managers, etc.) in a remote facility.
1.3.11. Administrator/director of hospital (institute): Hospital administrators/directors of local and remote facilities who have administrative responsibility for the overall practice including remote surgery (full telesurgery/telesurgical support/telementoring).
1.4. How to use the Guidelines.

These guidelines can be used as an overall map for practicing telesurgery in various clinical settings. These guidelines are intended to be cross-disciplinary in terms of organs treated and medical departments involved. In Japan, specific indications and procedures for telesurgery for various diseases should be in accordance with the guidelines established by the relevant academic societies specializing in those diseases.

2. Specific remarks 1: telesurgical support
2.1. Requirements for the overall provision of telesurgical support.
2.1.1. Requirements for medical teams providing telesurgical support.
2.1.1.1. Requirements for surgeons involved in telesurgical support.
2.1.1.1.1. Remote surgeon.

A remote surgeon must be certified as having sufficient skills to assist the local surgeon in safely performing telesurgery. In Japan, the remote surgeon must be a robotic surgery instructor/proctor certified by the Japanese Society of Endoscopic Surgery, the Japanese Society of Robotic Surgery, or other related societies, and must have completed a training program in telesurgery provided by the Japan Surgical Society or other related organizations.

2.1.1.1.2. Local surgeon

The local surgeon must have completed the training program given by the manufacturer of the surgical robot to be used, as the primary surgeon, and must also have obtained certification in robot-assisted endoscopic surgery from a relevant academic society. The local surgeon must have experience in performing planned surgeries or have received at least one direct training session by an instructor/proctor with certification from a relevant society. The local surgeon must have surgical experience in the respective field, equivalent to that of a board-certified specialist.

2.1.1.2. Requirements for operative staff involved in telesurgical support
2.1.1.2.1. Nurses

It is preferable that there be nurses present, in the role of direct assistants, at the local facility. These professionals should have completed a training program on the specific surgical robot being used or possess the equivalent, or higher, level of competence than mandated. Nurses are not, however, required at the remote facility.

2.1.1.2.2. Clinical engineers

Local and remote facilities must be staffed with clinical engineers who have completed the surgical robot manufacturer’s training program or have equivalent or better skills and experience in the maintenance and management of surgical robots. Such clinical engineers are expected to provide appropriate advice and troubleshooting for problems other than communication network issues that occur with the surgical robot. When participating as a direct assistant, it is essential that the clinical engineer have the appropriate level of knowledge and experience, as described above.

2.1.2. Fundamental requirements for medical facilities providing telesurgical support.
2.1.2.1. Requirements for remote and local facilities providing telesurgical support.
2.1.2.1.1. Local and remote facilities must have a medical safety management system as stipulated by law. In Japan, this corresponds to the Medical Care Act.
2.1.2.1.2. Local and remote facilities must be equipped with the type of communication network environment required for the stable delivery of telesurgical support. (See 2.1.3).
2.1.2.1.3. The local facility and the remote facility must prepare a communication environment (e.g., web conferencing system) that enables the remote surgeon to confirm the surgical environment at the local facility via video and to communicate with the local staff via voice in a bidirectional manner. The communication environment must use a separate line that is not affected by the communication line used by the remote surgery system.
2.1.2.1.4. Local and remote facilities must have a department (e.g., medical information department) that is responsible for the maintenance and management of the communication environment and other information infrastructure at the facility. The department must be familiar with the telecommunication environment necessary for telesurgery. They must work to maintain the telecommunication environment and cooperate with other facilities when implementing telesurgery.
2.1.2.2. Requirements for local facilities providing telesurgical support.
2.1.2.2.1. The local facility must be permitted to perform robotic surgery.

In Japan, the facility must meet the facility criteria for robot-assisted surgery by having received reimbursement for at least one such surgical procedure and must have notified the local health authorities of this. However, it is not necessary that the actual procedure for which telesurgical support is planned be included in the notified procedure.

2.1.2.2.2. The local facility at which telesurgical support is planned must have experience in performing at least one robotic surgical procedure at its own location. Such surgical experience is acceptable even if it was obtained through the proctoring system of the relevant society or its equivalent.
2.1.2.2.3. The local facility must have the necessary equipment and staff to complete the planned robotic-assisted surgery, as it may end up using alternative approaches such as open or endoscopic surgery, in cases where the telesurgical procedure becomes unfeasible to continue.
2.1.3. Requirements for the communication network environment for telesurgical support.
2.1.3.1. The communication network environment for telesurgical support must have the communication bandwidth required for stable operation of a surgical robot.

It is desirable to utilize lines with guaranteed bandwidth or assured forwarding that ensure the required bandwidth. If a so-called best-effort type line with variable available bandwidth is used, it must be confirmed in advance that communication bandwidth stably exceeds the bandwidth required for an operation where a surgical robot is to be used. Note that the required bandwidth varies depending on the model of the surgical robot, video compression method, etc.

2.1.3.2. It is necessary to confirm in advance that the communication line does not have large delays, significant jitter, or packet loss. Note that with best-effort lines, the communication environment at the time of surgery may differ significantly from that at the time of prior confirmation, as delay fluctuations and packet loss can change significantly from instance to instance.
2.1.3.3. The sum of the round-trip communication network transmission delay time and information processing delay time,* newly generated in the telesurgery environment, should be within 100 ms at the maximum.

*: Information processing delay time: time required to compress and decompress information signals for transmission and reception between remote locations.

2.1.3.4. The communication line must be capable of implementing the security measures described in “2.1.6 Security measures necessary for telesurgical support” below.
2.1.3.5. It is desirable to reduce the risk of communication network failure (communication line disconnection, communication network congestion, etc.) through a redundant communication line configuration. Redundancy of communication lines includes redundancy of communication line types as well as redundancy of communication carriers. In such cases, it is necessary to confirm in advance that surgical operations and the surgical robot system are not affected during line disconnection, line switching, or line switching back.
2.1.4. Requirements for surgical robots and devices for telesurgical support.
2.1.4.1. The surgical robot to be used must be a medical device approved by the accredited organization. In Japan, the surgical robot must be a medical device officially approved as a highly controlled medical device (Class III) in accordance with the “Act on Securing Quality, Efficacy and Safety of Products Including Pharmaceuticals and Medical Devices.”
2.1.4.2. The surgical robot must be approved for use in telesurgical support.
2.1.4.3. The surgical robot must have a published communication bandwidth commensurate to that required for stable operation. If the required bandwidth is variable, the range must be clearly indicated.
2.1.4.4. The surgical robot must have specifications that assume and adjust for instantaneous communication breakdowns, communication delays, and packet losses. In other words, the robot must have a function to stop control equipment or mitigate malfunctions in the event of instantaneous communication breakdown, delay, packet loss or packet disorder.
2.1.4.5. Devices directly attached to and used with the surgical robot must be those recommended, from a safety standpoint, by the manufacturer and the distributor of the robot.
2.1.4.6. The surgeon’s cockpit of the robot should have the ability to switch the operating privileges of the local and the remote surgeon and to record the history of any such changes.
2.1.5. Information security management system.

Surgeons, surgical staff, facility administrators, surgical robot manufacturers, and telecommunication carriers at both local and remote facilities need to take information security measures in accordance with laws, regulations, and the latest version of guidelines to ensure the availability of telesurgical support. In Japan, information security measures must be taken in accordance with the latest versions of the “Guidelines for Online Clinical Practice,” “Guidelines for the Safety Management of Medical Information Systems (https://www.mhlw.go.jp/content/10808000/001102570.pdf, in Japanese)”, and “Safety Management Guidelines for Providers of Information Systems and Services that Handle Medical Information (https://www.soumu.go.jp/main_content/000891033.pdf, in Japanese)”.

2.1.5.1. Surgeons and facility administrators at both local and remote facilities should have a basic knowledge of conducting telemedicine.

In Japan, it is mandatory for practitioners to attend online clinical practice training courses designated by the government. It is also desirable for local surgical staff and telesurgical staff to fully understand the content of the “Guidelines for Online Clinical Practice.”

2.1.5.2. Surgeons, surgical staff, and facility administrators at both local and remote facilities need to independently implement information security measures at their facilities in accordance with laws, regulations, and guidelines. In Japan, information security measures must be taken in accordance with the “Guidelines for Online Clinical Practice” and the “Guidelines for the Safety Management of Medical Information Systems.”
2.1.5.3. Surgical robot manufacturers and telecommunication carriers that provide a telesurgical environment should establish a system that enables safe implementation of telesurgical support in cooperation with local facilities and remote facilities, based on the laws, regulations, and guidelines. In Japan, it is necessary to establish an appropriate environment for telesurgical support based on the “Safety Management Guidelines for Providers of Information Systems and Services that Handle Medical Information.”
2.1.5.4. The contract or agreement between the local surgeon/institutional administrator and the remote surgeon/institutional administrator for the provision of telesurgical support must include a description on the information security management system as indicated in “2.1.6 Security Measures Required for Telesurgical Support.”
2.1.6. Security measures required for telesurgical support.

The integrity and confidentiality of information security must be guaranteed while ensuring the availability of telesurgical support. Specifically, the local and remote facilities need to take the appropriate measures for all aspects to do with maintaining the telesurgical support environment. This includes equipment-related measures, technical measures, organizational measures, such as instructions and accountability systems, and personnel measures, such as education and training.

2.1.6.1. Security measures for telecommunication lines.

The communication line used must be capable of taking the following security measures.

2.1.6.1.1. A closed communication network such as layer-3/layer-2 virtual private network (L3/L2VPN) that is physically or logically separated from the internet is desirable. When an open line is used for part of the network, or when an internet VPN line is used for the purpose of maintenance and management of the surgical robot, a highly secure method such as an IPSec + IKE (version 2) connection is recommended. At the same time, it is necessary to limit the destination/source IP addresses and port numbers used as much as possible by means of firewalls, etc.
2.1.6.1.2. Encryption between communication terminals is required. The same applies when connecting to an external line for the purpose of robot maintenance and management, etc.
2.1.6.1.3. When performing maintenance of surgical robots remotely from outside, the company entrusted with maintenance work needs to prepare a maintenance management plan detailing security compliance items such as access methods and authority management, depending on the nature of the maintenance work, and to establish rules and procedures for handling administrator authority and access, and obtain approval from the facility for such plan.
2.1.6.1.4. When internet VPN equipment is used, firmware must be kept up to date to ensure that it is not vulnerable; it should be noted that there is no zero-risk method for spoofing IP addresses.
2.1.6.1.5. It is essential that the intra-facility connection between the carrier’s line terminal and the surgical robot’s communication terminal be physically or logically separated from the intra-facility connection of other lines.
2.1.6.2. Security measures for surgical robots (including information processing terminals).
2.1.6.2.1. Communication encryption devices should be equipped between surgical robots or information processing terminals at the local facility and remote facilities.
2.1.6.2.2. Vulnerability information on the OS and middleware in the control system of the surgical robot must be constantly checked. If a vulnerability is found, measures such as applying a correction program (patch) must be taken as soon as possible according to the severity of the vulnerability.
2.1.6.2.3. It is necessary to clarify the setting of accessing privileges to the robot system, such as privilege IDs with access rights to set all parameters for the surgical robot, operation administrator IDs, robot operator IDs, etc., and to authenticate and authorize access to the minimum number of personnel deemed necessary for business purposes.
2.2. Implementation system for telesurgical support.
2.2.1. Preparatory procedures at the facility for the implementation of telesurgical support.

The local and remote facilities must have a system for providing and performing telesurgery as described in the Guidelines, and these systems must be approved by the safety management system of the facility (e.g., medical safety management committee, etc.). In Japan, local and remote facilities are required to obtain institutional approval of this technology as a “Highly Difficult New Medical Technology” as stipulated in the Medical Service Act before commencing telesurgical support. The measures required for using “Highly Difficult New Medical Technology” are obligatory for approval as an advanced treatment hospital from the government. Hospitals that do not fall into the category of an advanced treatment hospital can outsource a review to an external committee for evaluation as to their suitability to utilize “Highly Difficult New Medical Technology.”

2.2.2. Surgical procedures for which telesurgical support can be provided.

The surgical procedures that can be performed with telesurgical support are based on regulations as well as guidelines from the government and academic societies. In Japan, robotic surgery procedures that are covered by insurance as “surgery using endoscopic surgical support devices” are allowed to be performed as telesurgical support. However, it should be noted that surgical procedures that are accepted as covered by insurance differ depending on the model of the surgical robot. In addition, surgery must be performed in accordance with the guidelines and regulations stipulated by the relevant academic societies in each field.

2.2.3. Preparation for introducing telesurgical support.
2.2.3.1. Education of local surgeons and surgical staff.

It is recommended that local surgeons and local surgical staff observe actual telesurgical support at other facilities and use the experience to prepare for implementation at their own facilities. Local staff involved in telesurgical support should prepare an operation manual, hold regular conferences and study groups, and establish an adequate education system for staff. The manual must be shared among local and remote facility staff when telesurgical support is provided. The materials and educational program must also be reviewed as appropriate.

2.2.3.2. Preoperative review of cases.

The local surgeon, local surgical staff, and remote surgeon must hold a conference in advance to thoroughly discuss the appropriateness of providing telesurgical support (patient condition, indication, surgical procedure, etc.), as well as the division of roles between the local and remote surgeons, the possibility of changing the surgical procedure, and what to do if it becomes difficult to perform the procedure as a telesurgery. The details of these discussions must be documented in the patient’s medical record. When using a web conference system, the consent of the patient must be obtained in advance regarding the handling of the patient’s personal information; appropriate security management in compliance with relevant guidelines must be implemented.

2.2.3.3. Preoperative explanation to patients and obtaining consent.

When obtaining consent from a patient, the local surgeon or a physician on the local surgical site must provide the patient with an overview of telesurgical support, information about the remote surgeon, advantages of telesurgical support and possible disadvantages, in addition to general preoperative explanations, the following information is included.

2.2.3.3.1. The remote surgeon performs some surgical operations at a remote facility using a surgical robot.
2.2.3.3.2. The patient’s personal information and disease status are provided to the remote surgeon.
2.2.3.3.3. Even in the event of a malfunction of the information and communication equipment between the surgical robots, the local surgeon and local surgical staff can safely continue the ongoing surgery.
2.2.3.3.4. The local and remote facilities must take sufficient security measures to prevent unauthorized access by third parties and information leakage.
2.2.3.4. Pre-operational check of communication network and implementation environment.

The local facility and the remote facility must have prior approval from each facility’s safety management system (e.g., medical safety management committee) stating that the provision and implementation systems for telesurgery support indicated in the Guidelines are in place. The local and remote facility staff must confirm the communication network and implementation environment between the two facilities in advance using a checklist. The checklist must include the operational status of each of the surgical robots, the various devices, and the video and audio communication systems to be used between the local and remote facilities.

2.2.4. Ensuring safety during telesurgical support.

In preparation for emergencies during telesurgical support, a means of emergency communication between operating rooms at the local and remote facilities must be confirmed in advance. Local surgical staff and telesurgical staff must include clinical engineers who are familiar with the maintenance and management of the surgical robot to be used. In addition, a system must be secured to enable emergency contact with the person in charge at the manufacturer and distributor of the surgical robot. Information such as the date and time of telesurgical support must be shared with the department (e.g., Medical Information Department) responsible for the maintenance and management of the information infrastructure such as the communication environment at the facility. A system must be in place to contact the department promptly in the event of an error in the communication line during surgical support. In addition, it is desirable to have a system in place that enables urgent inquiries to telecommunication carriers regarding the lines.

2.2.5. Response to adverse events during telesurgical support.

Local/remote surgeons and local surgical staff should prepare and share an “Emergency Response Manual,” in advance, to deal with intraoperative emergencies. If a situation arises during telesurgical support that makes it impossible to perform the surgery properly or to ensure patient safety, the local surgeon or local surgical staff must take the initiative to promptly decide whether to suspend or cancel the tele-operation and proceed with the response in accordance with their “Emergency Response Manual.” The manual must include the criteria for decision to suspend or discontinue telesurgical support, as well as the criteria for conversion to open or thoraco/laparoscopic surgery. This manual must also be reviewed on a regular basis.

2.2.6. Change in a planned procedure during telesurgical support.

If a procedural change during telesurgical support is deemed necessary by a consensus of the local surgeon and the remote surgeon, telesurgical support may continue as long as the procedure has been previously authorized for performance in accordance with the applicable guidelines. If telesurgical support becomes difficult after the change, it must still be possible for the procedure to be completed by the local facility staff only.

2.3. Apportionment of responsibility for telesurgical support.

In principle, the local surgeon and the local facility administrator are responsible for the overall outcomes of the practices related to telesurgical support and postoperative complications. The local surgeon and the local facility administrator should thoroughly discuss the case in advance with the remote surgeon, and the remote facility administrator should determine whether or not responsibility for each case will be divided (if “yes,” the nature and extent of the division must be determined). The administrator must then prepare a written agreement or a written record in lieu of such an agreement.

2.4. Patient–physician relationship in telesurgical support.

The local surgeon must be treating the patient directly as the attending physician or equivalent. On the other hand, the remote surgeon may participate in telesurgical support without having been involved in prior direct patient care.

2.5. Cost sharing in telesurgical support.

Telesurgical support ensures standard or above-standard quality of care with the participation of a remote surgeon with specialized skills. In Japan, the inclusion and treatment of telesurgical support in the reimbursement system has not yet been determined, but it should be evaluated at least the same or better than the same procedure performed in conventional robot-assisted surgery. In addition to the surgical costs incurred locally, telesurgical support incurs supplemental costs for personnel, facilities, and equipment at the remote facility where the surgical support is provided, as well as communication line costs. In particular, the fees for guaranteed bandwidth lines with a high level of communication security are expensive at present, and the use of more economical lines and methods of bearing the high line costs are issues to be addressed in the future.

From the perspective of expanding the number of sites supported by telesurgery, it is desirable to use virtual leased circuits that allow multipoint connections. It is also necessary to promote the emergence of economical communication services that guarantee the minimum bandwidth required for operation of surgical support robots. Considering that telesurgical support is essentially operated from a humanitarian perspective, targeting elderly patients living in remote areas who have difficulty moving, it is also important to consider making it eligible for public subsidies.

3. Specific remarks 2: telementoring

In these Guidelines, telementoring is defined as the use of images and audio by a telesurgical instructor to guide the local surgeon in real time, regardless of the surgical procedure, e.g., robot-assisted surgery, thoraco/laparoscopic surgery, or open chest/abdominal surgery.

3.1. System for providing telementoring.
3.1.1. Requirements for a communication network environment to provide telementoring.
3.1.1.1. The communication environment used for telementoring varies greatly depending on the amount of video information to be transmitted, the required stability and completeness of communication, real-time performance, and economic efficiency. Therefore, it is necessary to construct and implement an appropriate communication environment according to the specific demands of the telementoring.
3.1.1.2. Open lines with internet connection or so-called best-effort lines are lines where communication delay, delay fluctuation, and packet loss can change from moment to moment. It should be noted that the communication environment during actual telementoring may differ significantly from that of prior confirmation.
3.1.2. Information security measures required for telementoring.

When handling patient identifiers, telementoring staff, local surgeons, local surgical staff, and local facility administrators must take appropriate information security measures. In Japan, information security measures must be adhered to in accordance with the latest version of the “Guidelines for the Secure Management of Medical Information Systems” along with the information security policies of the local and remote facilities.

3.1.2.1. Contractors that provide a telementoring environment must take appropriate information security measures because they are dealing with sensitive data, including the patient’s personal identification information related to medical care. In Japan, measures should be taken in accordance with the “Guidelines for the Safety Management of Information Systems and Services Providers that Handle Medical Information” and the information security policies of the local and remote facilities.
3.1.2.2. When telementoring is provided as part of telesurgical support, or when surgical guidance requires high real-time performance, information security measures need to be taken in accordance with those mandated for telesurgical support.
3.1.2.3. Even when patient information (personally identifiable information) related to medical care is not handled, it is necessary to take measures to protect all personal information, such as measures to prevent the inclusion of personal information of staff in operating room videos.
3.1.2.4. When performing telementoring using advanced equipment such as VR, it is necessary to constantly check the vulnerability information of the OS, middleware, and firmware of the system concerned. If a vulnerability is discovered, it is imperative to take measures such as applying a correction program (patch) as soon as possible according to the severity of the vulnerability. In addition, appropriate subject authentication and authorization settings must be created for each user and maintenance operator of the telementoring system. The number of users should be kept to the minimum necessary for successful completion of tasks, and should be in accordance with the actual conditions of use.
3.2. Implementation of telementoring.
3.2.1. Procedures for which type of telementoring can be provided.

All robotic procedures can be performed under telementoring. In Japan, surgeries reimbursed by national health insurance are eligible for telementoring.

3.2.2. Preparation for implementation of telementoring.
3.2.2.1. Prior review of the case.

The local surgeon and the remote instructor must thoroughly review the details of the tele-operative guidance for the treatment of the patient and must document them in the local facility’s medical records. The patient’s consent regarding the handling of the patient’s personal information must be obtained prior to the review with the remote instructor.

3.2.2.2. Explanation to the patient and consent acquisition.

When obtaining consent from the patient, the patient should be given an overview of what telementoring for the procedure entails, including information about the telementoring instructor, as well as the advantages and possible disadvantages of telementoring, in addition to a general preoperative explanation.

3.2.2.3. Prior confirmation of the communication and implementation environment.

The local surgeon, local surgical staff, and remote instructor must confirm the communication environment between the two facilities and the operational status of the devices to be used in advance.

3.2.3. Response to adverse events during telementoring.

If a situation arises during telementoring in which the proposed surgery cannot be performed properly or will make it difficult to ensure patient safety, the local surgeon or local surgical staff must take the initiative and promptly make the decision to suspend or discontinue the telementoring.

3.3. Apportionment of responsibility for telementoring.

In principle, the local surgeon and the local facility administrator are responsible for medical practices and results related to telementoring. However, the specific details should be thoroughly discussed beforehand, and a written agreement or alternative record should be prepared.

3.4. Patient–doctor relationship in telementoring.

The remote instructor may provide telementoring without providing medical care to the patient. In Japan, telementoring is categorized as a form of D to D in the “Guidelines for Online Clinical Practice.”

4. Specific remarks 3: full telesurgery

Full telesurgery assumes that a remote surgeon performs all surgical procedures from a remote facility using a surgical robot when there is no physician available at the local facility to perform the operation. Therefore, it is very different from telesurgical support, in which the operation is continued by the local surgeon and local surgical staff even if the telesurgery is interrupted. An extremely high level of certainty and safety must be ensured in the implementation of full telesurgery.

In addition, Article 20 of the Medical Practitioners Law in Japan prohibits medical practices that do not involve direct consultation, but online clinical practices that resemble direct consultation are permitted if they are included in the Guidelines for Online Clinical Practice. Therefore, as a prerequisite for implementation, full telesurgery must be stipulated in the “Guidelines for Online Clinical Practice.” Due to these legal issues, as well as many other technical and ethical issues, it is difficult to implement full surgery in Japan at this time. It is expected that with the innovative progress of surgical robots and information and communication technology, social implementation will become possible in the future.

Committee members: Masaki Mori (Japan Surgical Society/School of Medicine, Tokai University), Satoshi Hirano (Department of Gastroenterological Surgery II, Hokkaido University), Kenichi Hakamada (Department of Gastroenterological surgery, Hirosaki University), Eiji Oki (Department of Surgery and Science, Graduate School of Medical Sciences, Kyushu University), Shigeo Urushidani (Information Systems Architecture Science Research Division, National Institute of Informatics), Ichiro Uyama (Advanced Robotic and Endoscopic Surgery, Fujita Health University), Masatoshi Eto (Department of Urology, Graduate School of Medical Sciences, Kyushu University), Yuma Ebihara (Department of Gastroenterological Surgery II, Hokkaido University), Kenji Kawashima (Graduate School of Information Science and Technology, The University of Tokyo), Takahiro Kanno (RIVERFIELD Inc.), Masaru Kitsuregawa (National Institute of Informatics, The University of Tokyo), Yusuke Kinugasa (Department of Gastrointestinal Surgery, Tokyo Medical And Dental University), Junjiro Kobayashi (National Cerebral and Cardiovascular Center), Hiroshige Nakamura (Department of General Thoracic Surgery and Breast and Endocrine Surgery, Tottori University), Hirokazu Noshiro (Department of Gastroenterology and General Surgery, Saga University), Masaki Mandai (Department of Obstetrics and Gynecology, Kyoto University), Hajime Morohashi (Department of Gastroenterological Surgery, Hirosaki University),

Advisors: Ryuichi Yamamoto (Medical Information System Development Center), Takafumi Ochiai (Atsumi & Sakai Legal Professional Corporation), Atsushi Kajitani (Kajitani Law Offices), Yusuke Tsugawa (University of California, Los Angeles),

Supervisors: Norihiko Ikeda (Japan Surgical Society), Yoshiharu Sakai (Japan Society for Endoscopic Surgery/ Osaka Red Cross Hospital), Koshi Mimori (Department of Surgery, Kyushu University Beppu Hospital), Go Watanabe (Japan Robotic Surgery Society/NewHeart Watanabe Institute),

Collaborators: Akira Endoh (Division of Medical Information Planning, Hokkaido University Hospital), Kuriko Kudo (Telemedicine Development Center of Asia, Kyushu University Hospital).
